# MicroRNAs as Potential Graft Rejection or Tolerance Biomarkers and Their Dilemma in Clinical Routines Behaving like Devilish, Angelic, or Frightening Elements

**DOI:** 10.3390/biomedicines12010116

**Published:** 2024-01-05

**Authors:** Isabel Legaz, Víctor Jimenez-Coll, Rosana González-López, Marina Fernández-González, María José Alegría-Marcos, José Antonio Galián, Carmen Botella, Rosa Moya-Quiles, Manuel Muro-Pérez, Alfredo Minguela, Santiago Llorente, Manuel Muro

**Affiliations:** 1 Department of Legal and Forensic Medicine, Biomedical Research Institute of Murcia (IMIB), Regional Campus of International Excellence “Campus Mare Nostrum,” Faculty of Medicine, University of Murcia (UMU), 30100 Murcia, Spain; 2 Immunology Service, University Clinical Hospital “Virgen de la Arrixaca”—IMIB, 30120 Murcia, Spain; 3 Service of Nephrology, Unit Hospital Clinic Universitario Virgen de la Arrixaca, IMIB-Arrixaca, 30120 Murcia, Spain

**Keywords:** allograft rejection, biomarker, chronic kidney disease, microRNAs, tolerance

## Abstract

Allograft rejection is a widespread complication in allograft recipients with chronic kidney disease. Undertreatment of subclinical and clinical rejection and later post-transplant problems are caused by an imperfect understanding of the mechanisms at play and a lack of adequate diagnostic tools. Many different biomarkers have been analyzed and proposed to detect and monitor these crucial events in transplant outcomes. In this sense, microRNAs may help diagnose rejection or tolerance and indicate appropriate treatment, especially in patients with chronic allograft rejection. As key epigenetic regulators of physiological homeostasis, microRNAs have therapeutic potential and may indicate allograft tolerance or rejection. However, more evidence and clinical validation are indispensable before microRNAs are ready for clinical prime time.

## 1. Background

The standard method for identifying organ rejection is typically the kidney allograft biopsy [1]. However, this procedure is invasive and can be unclear or inconclusive in some cases. Recent advancements have led to the discovery of successful kidney allograft rejection biomarkers, some of which are now available for routine clinical use [2,3,4]. An intriguing aspect of organ transplantation evolution and outcome involves monitoring the expression of key immune molecules that play a role in the biological response against donor allografts [5,6]. MicroRNA (miRNA) is one such regulatory element that can determine, modulate, or influence the expression of these molecules [7]. Previous research has implicated miRNAs in transplant outcomes [8,9,10,11,12], and this study explores their role in both pre- and post-transplant monitoring. 

This systematic review includes studies spanning 17 years (2006–2023) that examine human microRNAs in graft rejection or tolerance. This study covers four main areas: (i) miRNA and biological functions, (ii) microRNAs as circulating biomarkers in health and disease, (iii) miRNA molecules and their role in transplantation, and (iv) miRNA molecules in B cell, humoral rejection, and DSA production.

**Figure 1:**
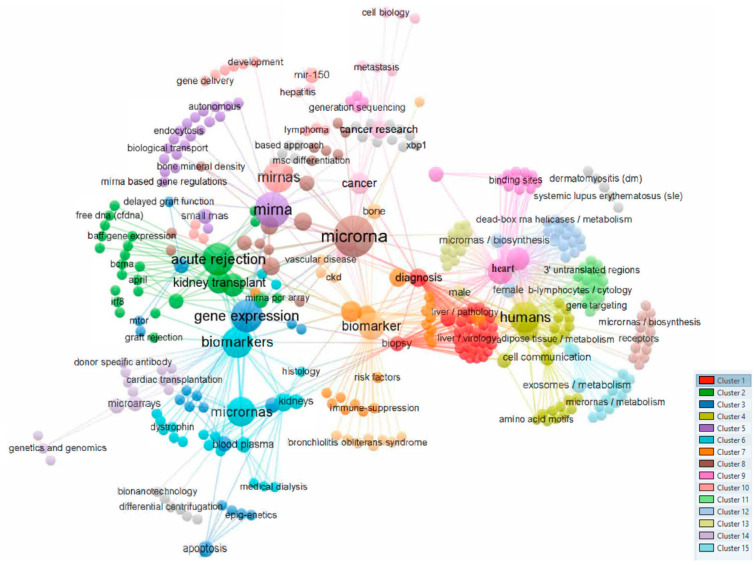
Analysis of co-occurrences in the top 537 keywords.

Figure 1 shows a bibliometric analysis based on the co-occurrence of all keywords as the unit of analysis was performed using VOS viewer [13], visualizing a total of 14 different clusters (Figure 1).

Currently, different studies hypothesize that microRNA (miRNA) molecules play crucial roles in various biological processes, including transplantation, and that understanding their regulatory functions can contribute to predicting and understanding graft outcomes. It is suggested that miRNAs, which regulate gene expression at the post-transcriptional level, can serve as valuable biomarkers in health and disease, particularly in organ transplantation.

The aim of this systematic review was to comprehensively explore the role of microRNA (miRNA) molecules in fundamental biological functions, such as cell cycle, differentiation, proliferation, and cell death. The genesis and functionality of miRNAs were delved into. On the other hand, the potential of miRNAs as circulating biomarkers in health and disease was analyzed, emphasizing their stability and resistance to freezing and thawing cycles. We delved deeper into miRNAs in kidney allograft transplantation, aiming to reveal insights into predicting graft status. The analysis was extended to the involvement of miRNAs in B cell function, humoral rejection, and donor-specific antibody (DSA) production during transplantation. The challenges and opportunities associated with the analysis of circulating miRNAs were also evaluated and miRNA expression profiles were evaluated, considering their potential as diagnostic and prognostic markers in transplants. Finally, the main limitations and challenges in miRNA studies were addressed.

## 2. MicroRNA Molecules and Biological Functions

RNA molecules transcribed from DNA include different types, such as RNA messenger (mRNA) or miRNAs. miRNAs, a class of non-coding RNAs, play a crucial role in post-transcriptional regulation, affecting processes such as cell cycle, differentiation, proliferation, and cell death. 

The study of miRNAs, which are non-coding endogenous RNA molecules with regulatory and modulatory roles that prevent target mRNAs from being translated and have a length of 20–24 nucleotides, is ongoing [11]. Different studies estimate that these molecules could regulate approximately 60% of the human transcriptome [9,10,14]. Cell cycle, differentiation, proliferation, and cell death are only a few of the critical biological processes that their post-transcriptional repression of significant and determinant mRNA targets regulate. The development of high-throughput sequencing technology [14] and predictive computational and bioinformatics tools [15] has considerably expanded the research of miRNAs, including their putative regulatory targets and biological functions.

In brief and functionally, miRNAs bind to the complementary mRNA’s 3′-UTR region, inhibiting protein translation or promoting mRNA degradation [11]. Most miRNA sequences are found in non-coding RNA molecules’ introns or exons. However, some miRNAs (known as mirtrons) come from introns in pre-mRNA. As previously reported, different miRNA localizations determine canonical or non-canonical pathways [16]. Numerous miRNAs play roles in various biological processes, such as organ transplant rejection, hematopoietic stem cell transplantation, neural patterning, fat metabolism, cell death, proliferation, and differentiation [17,18,19,20,21].

A crucial implication is that one miRNA can target hundreds of mRNAs, and vice versa; several miRNAs can target one mRNA [19]. Consequently, genetic expression is fine-tuned via a complex network of miRNAs and mRNAs. The fact that patterns of miRNA expression appear to be tissue-specific and substantially conserved across species emphasizes the significance of these molecules for evolution [20,22]. Over half of the 30,000 or so human mRNA transcripts are regulated by the 2600 mature miRNAs that have so far been found in the human genome (miRBase V22) [23].

Dysregulation of particular miRNAs is published in and may enhance or direct oncological, inflammatory, autoimmune, metabolic, or neurodegenerative diseases [21,24,25,26]. In addition to being essential intracellular regulators of gene expression, miRNAs can have paracrine and endocrine effects after being actively taken up by cells [27,28]. miRNAs have recently been identified as interorgan circulating communication mediators [29,30,31]. In the setting of kidney allograft transplant, it is crucial to notice that hemodialysis does not eliminate circulating miRNAs, as previously reported [32].

**Figure 2:**
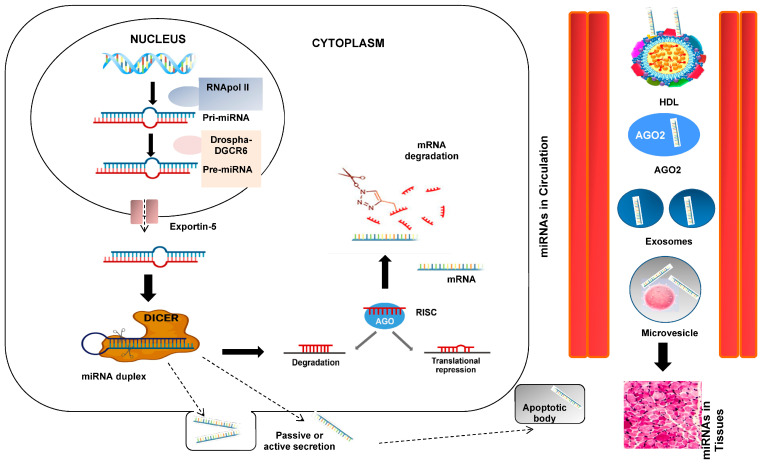
miRNA biogenesis and secretion. The primary miRNA (pri-miRNA), which is several kilobases long and contains regional stem-loop structures, is produced in the nucleus from miRNA-encoding genes by RNA polymerase II. Precursor miRNA (pre-miRNA) is the name given to the hairpin structure that is produced when pri-miRNA is cut at the stem by the RNase III enzyme Drosha and its cofactor DiGeorge Syndrome Critical Region 8 (DGCR8). A second RNase III enzyme called DICER cleaves the pre-miRNA into the double-stranded miRNA after being delivered to the cytoplasm by exportin-5. The Argonaute 2 protein chooses the miRNA strand with the less thermodynamically stable 5′ end to be the guide strand and integrates it into the RNA-induced silencing complex (RISC). The RISC weakens the passenger strand and the other strand. Based on the sequence-specific binding of 5–7 complementary nucleotides in the 3′-UTR region, the mature miRNA in the RISC binds to its target mRNA. This binding facilitates the inhibition of translation or the breakdown of mRNA. Interesting steps in the mediating effects of miRNAs include passive or active miRNA production, miRNAs in blood circulation, and miRNA entry into tissues.

At this point of our introduction, we could appropriately ask about the genesis and function of these interesting miRNAs. To clarify and understand this process, a figure showing the genesis processes is shown (Figure 2). RNA polymerase II or III transcends miRNA to primary miRNA (pri-mRNA). pri-miRNA is cleaved by the Drosha-DGCR8 microprocessor complex in the core. The resulting precursor pre-miRNA is exported to the nucleus by Exportin-5-Ran-GTP. In the cytoplasm, the RNase Dicer in complex with the double-stranded RNA-binding protein TRBP cuts the pre-miRNA at its mature form [16]. The mature miRNA is loaded together with the Argonaut (Ago2) protein into the RNA-induced silencing complex (RISC), where it guides the RISC to the target mRNA, causing its repression by mRNA cleavage, translational repression, or deadenylation [10,11].

Currently, mature miRNAs can either travel from the cell cytoplasm into extracellular circulation or detect target mRNAs and cause mRNA silencing. It has also been noted that extracellular miRNAs are more stable than cellular miRNAs [33]. miRNAs circulating or cell-free in physiological fluids have recently been studied and found to be potential biomarkers for some pathological conditions, including diagnosis, prognosis, and cancer therapy. [34]. This is a vast and exciting current and future research area. Extracellular miRNAs can be transported in vesicles such as exosomes, apoptotic bodies, and microvesicles in addition to their interactions with proteins, particularly AGO2 [35]. Exosomes may also include mature or pre-miRNA forms since some cancer exosomes have a protein processing complex (RISC-loading complex), indicating cell-independent miRNA maturation [36].

Exosomes have historically served as transporters to the local microenvironment and a vital communication channel between cells and tissues. They may be taken up by recipient cells, and subsequently released miRNAs may cause changes in the expression of significant target genes [37]. Exosomal miRNAs have been adequately implicated in the pathophysiology and progression of cancer, according to studies, and they have the potential to be used clinically [37].

Table 1 shows a summary of the main findings on microRNA molecules and their biological functions.

## 3. MicroRNAs as Circulating Biomarkers in Health and Disease

As previously indicated, microRNAs can be present in biofluids, packaged inside extracellular vesicles (exosomes and microvesicles), or bound to lipoproteins and ribonucleoproteins. Previous studies have shown that active cellular transport routes mediate the selective secretion and absorption of miRNAs, which determines the composition of circulating miRNAs. However, the varied pool of extracellular miRNAs is also influenced by the passive leakage of miRNAs from damaged or dead cells [38,39,40,41,42,43].

Circulating miRNAs are great candidates for significant clinical biomarkers due to some characteristics of miRNAs. As carriers shield them from endogenous RNases, microRNAs are stable in peripheral blood and can be successfully detected in samples kept for a considerable amount of time. Also, miRNAs are believed to resist repeated freeze–thaw cycles [33,44,45,46].

Another critical point is that circulating miRNAs represent tissue-specific expression levels, as reported [47,48,49,50,51].

How can we quantify these molecules? Standard techniques for detecting and quantifying specific individual miRNAs include several approaches. Simple, sensitive RT-qPCR assays, Northern blot, and in situ hybridization can be used to measure microRNAs quantitatively. In contrast, common methods for the global profiling of miRNAs such as high-throughput methods like micro-arrays, TaqMan low-density arrays, counter miRNA expression assay, and RNA sequencing that can screen for specific miRNAs of interest or quickly analyze the patterns of larger miRNA panels with more precise computational risk prediction, diagnoses, treatment advice, monitoring, and outcomes in blood, cells, or tissues are made possible by quick and affordable multi-miRNA panels.

miRNAs have been identified as a novel class of very sensitive circulating biomarkers for numerous metabolic and age-related disorders, as evidenced by the advancement of this field’s research over the past ten years [41]. Licensed diagnostic miRNA panels are available for cardiovascular illness, Alzheimer’s disease, thyroid, pancreatic, and breast cancer [52].

Even some of these panels have the backing of well-known insurance companies. For instance, osteomiRTM, a panel of 19 plasma miRNAs, has been suggested as a risk predictor for osteoporotic fractures in postmenopausal women independent of bone mineral density (BMD) [53]. Several pools of miRNAs have also been proposed as biomarker illnesses.

It is also necessary to mention the difficulties encountered when analyzing circulating miRNAs. Preanalytical errors are to blame for most errors in a clinical chemistry lab. Controlling the preanalytical variability of biospecimens is crucial since it can considerably impact subsequent studies [54]. This is true for circulating miRNAs as well. Essential patient features, including age, sex, body size, and variations brought on by the circadian rhythm, diet, or lifestyle, may impact how they are expressed or modulated.

With more time spent on the bench before processing, there is a greater chance of hemolysis or blood cell leakage, which could change miRNA expression. To reduce the variability of miRNAs, it is crucial to standardize the collection, processing, transport, and storage processes. Variability in analytical results should also be taken into account. Different normalization techniques and the heterogeneity of the commercially available tests reduce reproducibility and lead to ambiguous results. [55,56,57,58]. In this sense, the correct and mandatory standardization of miRNA testing in clinical laboratories and confounding elements should be thoroughly established and revised.

Several articles have recently shown miRNAs as a new biomarker of inflammation in inflammatory bowel disease (IBD) [59,60,61]. In this sense, Abdelazim et al. 2023 explain that serum miR-486 could be employed in the risk stratification of IBD subtypes and has grounds for clinical utility in Crohn’s disease (CD) diagnosis, whereas miR-25 has the potential for ulcerative colitis (UC) and CD diagnosis.

Finally, miRNAs reduce/increase proliferation and metastasis and modulate cell death and proliferation. The overexpression of oncogenic miRNAs facilitates drug resistance and radio-resistance in lung cancer. Tumor microenvironment components, including macrophages and cancer-associated fibroblasts, demonstrate interactions with miRNAs in lung cancer. Other factors such as HIF-1α, lncRNAs, and circRNAs modulate miRNA expression. miRNAs also have value in the diagnosis of lung cancer [62].

Table 2 shows a summary of the main findings on microRNAs as circulating biomarkers in health and disease.

## 4. MicroRNA Molecules and Their Role in Transplantation

The role of miRNAs in kidney transplantation begins, particularly in knowing that prior to transplantation, they can play a crucial role in nephrological pathologies that give rise to the poor function of the healthy kidney, which can lead to needing a transplant. In this way, miRNAs seem to play a role in an extensive catalog of urinary tract pathologies that cover various tumors, infectious pathologies, systemic autoimmune pathologies with renal involvement, and isolated renal pathologies related to the immune system, which can cause native kidney damage and that could presumably reappear after kidney transplantation. miRNAs appear to regulate all these processes, pathologies, evolution, and results. It is known and inferred that different changes in miRNA expression could modify or modulate kidney transplantation’s short- and long-term evolution. The vast majority of studies on kidney allograft transplantation that direct or analyze the role of miRNAs or mRNA detection and expression have been conducted with blood samples (isolating cells as peripheral blood leukocytes or separating plasma or serum) or urine samples, and a small percentage of studies have analyzed the expression of intra-graft miRNAs comparing their eventual correlation with the levels of the rest of the mRNA [5,6,8,63]. RNA quantification indirectly measures the gene expression levels in a person’s blood, cells, fluids, or tissues.

Several articles from recent years emphasize the function of miRNA expression in solid organ transplantation and imply that it has a role in the acceptance or rejection of allografts [21,63].

Additionally, as previously reported [64,65], the analysis of miRNA expression profiles has shown promise for predicting kidney graft status in various clinical contexts, such as acute rejection or delayed graft function. However, these findings must be fully verified in a patient series with a larger patient population.

In this sense, the analysis transcriptomics can help discover expression profiles that allow not only to stratify patients according to immunological risk to rejection or tolerance but also to differentiate the type of rejection when this occurs, providing valuable information to the clinician concerning modifying the treatment of a particular patient [66].

On the other hand, these molecules could also have an essential function in cold ischemia-reperfusion injury, as previously reported [67,68]. Additionally, since their discovery, research on miRNA expression profiles has shown promise for predicting kidney graft status in a variety of clinical contexts, including acute or delayed graft function, rejection, liver and heart transplant, hematological transplantation, or bone fragility in chronic kidney disease [6,26,69,70,71,72,73,74,75].

In this sense and with more detail in these facts, a research group examined urinary exosomal microRNAs to identify novel biomarkers of rejection [69]. Candidate microRNAs were selected using NanoString-based urinary exosomal microRNA profiling, meta-analysis of a web-based, public microRNA database, and a literature review. They identified 29 urinary exosomal microRNAs as candidate biomarkers of acute rejection, of which seven were differentially expressed (DE) in recipients with acute rejection. A three-microRNA acute rejection signature, composed of hsa-miR-21-5p, hsa-miR-31-5p, and hsa-miR-4532, could discriminate recipients suffering acute rejection. Other authors also provide a summary of information about the therapeutic relevance of aberrant miRNA expression profiles in hematologic cancer patients and their relationships with various antagomiRs, mimetics, and circular RNAs (circRNAs), as well as with diagnosis, prognosis, and therapy response monitoring [70]. A narrative review written by a different group also examined whether miRNAs could aid in treating osteoporosis and renal osteodystrophy [26].

On the other hand, the group of Coutance et al. [73] also designed a prospective study (NCT02672683) including recipients from 11 centers between 08/2016 and 03/2018 [71]. The objective was to validate the association between three previously identified circulating microRNAs (10a, 92a, 155) and rejection. Overall, 461 patients were included in this study, and the results did not show the clinical utility of circulating microRNAs 10a, 92a, and 155 monitoring in heart allograft recipients.

In a separate investigation, kidney transplant recipients who developed acute rejection episodes had higher expression levels of six specific miRNAs (miR-191-5p, miR-223-3p, miR-346, miR-423-5p, miR-574-3p, and miR-181d), while miR-150-5p expression had decreased. The following analysis revealed a potential connection between miR-150-5p and transcription factor MBD6, suggesting that the modification of this interaction may be responsible for the beginning or development of acute rejection episodes [8]. More studies will be necessary to confirm these points.

In this way, miR-150 plays a crucial role in immune regulatory processes such as B and T lymphocyte proliferation, activation, and apoptosis. It is also explicitly expressed in lymph nodes, the spleen, and mature B and T cells [71,76]. One of the most researched miRNAs, it is involved in innate and adaptive immune responses and is crucial for the pathogenesis of various malignancies [75,76]. By controlling mTOR expression, miRNA-150 is also involved in Treg cell development and has been considered a lymphocyte activation biomarker [77,78]. These researchers established a correlation between an increase in serum and a decrease in miR-150 at the intracellular level following CD4+ cell activation.

Additionally, this study [78] proposed a new mechanism for regulating miR-150 intracellular levels, the expression of the genes it targets, and the genes required for immune activation, where the activation of CD4+ cells may be associated with a decrease in miR-150-5p. The expression of miR-150-5p was shown to be reduced in KTRs with AR in our sample, although earlier studies of a similar kind discovered that miR-150 was raised in people with ACR compared to biopsies without rejection [79]. However, different findings in biopsies or peripheral blood mononuclear cells were not found in other trials [80]. In conclusion, further research is needed before miR-150 can be posited as a viable biomarker in solid organ transplantation [81]. Other miRNAs implicated in rejection are the miR-181 family, which has been reported to increase in patients with acute kidney rejection [8]. Acute renal injury brought on by ischemia-reperfusion has been linked in mouse models to a decrease in miR-181d expression [82]. There is no information on the role of miR-181d in humans, even though other miRNAs from the miR-181 family, such as miR-181a, have been linked to acute rejection in kidney transplantation. The development of B and T cells is also differentiated and activated by this miRNA [83]. On the other hand, miR-191 also showed overexpression in kidney acute rejection [8]. In lung transplantation, the overexpression of miR-191 has been associated with bronchiolitis obliterans (BOS), which is the most common form of chronic lung transplant rejection [84]. In hematopoietic transplantation, this similar fact has also been associated with patients with a high mortality risk [85]. The main target genes for miR-191 are cell cycle regulatory transcription factors, such as CDK6, and chromatin remodelers, such as MDM4 [86]. Therefore, the deregulation of miR-191 could be associated with alterations in lymphoid populations and linked to rejection events.

Several studies show that miR-233 overexpression could also be associated with acute rejection or as a tolerance biomarker in renal graft biopsies [64,80]. Their overexpression during AR appears to not be specific to kidney transplants since similar results have also been obtained in murine liver transplant models [87,88]. Other studies report that the overexpression of miR-223 is associated with a worse kidney function during the first few weeks post-transplant [89].

The increase in miR-346 has also been associated with kidney rejection [8,90], and in heart transplantation, it has been correlated with the severity of ischemia-reperfusion damage [91]. The functions of miR-346 have been associated with the control of cell proliferation and regulation of TNF-α secretion, whose dysregulation has been linked to cancer and rheumatoid arthritis [88] and a modulator of HLA class I expression in the HeLa cell line by inhibiting the expression of the TAP1 gene [90]. Theoretically, the increase in miR-346, through the inhibition of Bcl-6, could favor a decrease in the allospecific responses mediated by antibodies by decreasing the levels of follicular T lymphocytes [92].

In addition, another miRNA, miR-423-5p, has been linked with graft damage in KTRs [91], and increased miR-574-3p has been associated with delayed graft function in kidney recipients [8], although other studies did not confirm these data [64]. Although these variations were not seen when compared to biopsies with AMR, miR-574 was reduced in liver recipients experiencing rejection in other investigations [79]. miR-574-3p adversely impacts the IL-6/STAT3 pathway, as demonstrated by other researchers [93]. Therefore, increased pro-inflammatory pathways resulting from STAT3 activation [93] may cause a decrease in miR-574-3p expression in samples with T-cell-mediated AR [79].

Finally, other important studies with respect to miRNA expression changes, in transplant complication and outcome, are the following: (a) miR-15a, miR-16, miR-103a, and miR-107 down-regulation has been implicated in T-cell-mediated rejection in PBMC [94]; (b) miR-21 up-regulation, in PBMC and tissue [95,96], has been associated with interstitial fibrosis tubular atrophy (IFTA); (c) miR-30c-5p has been associated with ischemia-reperfusion [97]; (d) miR-142-3p up-regulation has been associated with acute rejection, IFTA, and immunosuppressive drugs withdrawal [95,96,98]; (e) miR-142-5p up-regulation, in PBMC and tissue, has been associated with AR and IFAT [64,80,98,99]; (f) miR-155 up-regulation, in PBMC and tissue, has been associated with AR and IFAT [64,96]; (g) miR-211 down-regulation, in PBMC and tissue, has been associated with IFAT [80]; (h) miR-221 up-regulation, in PBMC and tissue, has also been associated with IFAT [95]; (i) miR-223 up-regulation, in PBMC and tissue, has been associated with AR [64]; (j) miR-494 has been associated with nephrotoxicity [98]; (k) miR-682 has been associated with extracellular vesicles from dendritic cells [100]; (l) Let-7d, miR-29, miR-30, miR-130, miR-186, miR-192, miR-200, and miR-109 have been associated with nephrotoxicity and immunosuppression [101]; and (m) let-7i has been associated with bacterial infection in cyclosporine-treated patients [102].

Thus, in conclusion, the role of miRNAs in organ transplantation needs more continuous and exhaustive research and validation in multiple cohorts and more studies to reach their complete establishment in clinical routines.

Table 3 shows a summary of the main findings on microRNA molecules and their role in transplantation.

## 5. MicroRNA Molecules in B Cell, Humoral Rejection, and DSA Production

We will discuss and deepen several exciting points regarding different miRNAs’ eventual role or influence in donor-specific antibody (DSA) production in transplantation.

Firstly, transcriptomics studies in tolerant recipients have shown increased expression of important genes related to B lymphocytes, such as CD79B or CD20 [103]. Other studies have accordingly shown that the decreased expression of CD79B, CD20, and TCL1A is associated with patients suffering acute rejection episodes [104] and the increased expression of cytokines such as CXCL9 and CXCL10, produced by B lymphocytes after their interaction with T lymphocytes, is present in rejection processes in various types of solid organ transplantation [93].

Numerous B lymphocyte subpopulations, including germline naive B lymphocytes, have had their expression of miRNAs examined, demonstrating that these molecules may be crucial for effector activities and regulatory networks governing cell growth [63,71]. In this manner, different miRNAs have been implicated in B cell function and antibody production.

Firstly, miR-150-defective animals have been shown to secrete more antibodies in response to antigenic stimulation [105]. Secondly, the abnormalities in miR-155 lead to an isotype change deficiency and poor plasma cell development [99].

Thirdly, another study by other authors revealed that B cells from tolerant kidney transplant recipients overexpressed the miR-142-3p gene [106], pointing to a potential mechanism involving the TGF-signaling pathway.

On the other hand, research on miRNA expression in B lymphocytes, including naive and germinal center B lymphocytes, has demonstrated that these molecules can be crucial in the regulatory pathways of cell growth and effector functions [107,108].

For instance, miR-150-deficient animals secrete more antibodies in response to antigenic stimulation [105,109]. The overexpression of miR-142-3p and miR-142-5p in B cells from tolerant kidney transplant recipients and patients with chronic antibody rejection, respectively, has been demonstrated by other authors in some articles, suggesting a potential mechanism involved in the opposing effects of the TGF-signaling pathway [106,110,111,112,113].

Finally, other critical studies in kidney transplantation concerning miRNA expression changes in clinical AMR complications in humans are the following: let-7c, miR-28, miR-30d, miR-99b, miR-125a, miR-195, miR-374b, miR-484, miR-501, and miR-520c up-regulation, and miR-29b and miR-885 down-regulation have been associated with AMR [103].

Table 4 shows a summary of the main findings on microRNA molecules in B cell, humoral rejection, and DSA production.

## 6. MicroRNA Molecules in Viral and Bacterial Infection in Transplantation

Kidney transplant recipients exhibit a series of infectious complications post-transplant that can be pretty common and have to do with urinary complications.

For example, in the case of bacterial infections, we have gastrointestinal tract infections caused mainly by uropathogenic *Escherichia coli* associated with the immunosuppressant cyclosporine. This will activate the cells that are a preferential site of adhesion and translocation for this specific pathogen, inducing the inhibition of the lipopolysaccharide-induced activation of the cells and down-regulating the expression of TLR4 through the miRNA let-7i. Furthermore, using an anti-let-7i during renal treatment can protect patients treated with cyclosporine from bacterial infections [114].

Concerning viral infection, we will name several examples common in kidney transplants. Firstly, Epstein–Barr viral miRNA profiles were observed in pediatric kidney transplant patients infected with EBV but overcoming this infection or chronically high viral loads compared to children without transplants and with acute infectious mononucleosis (IM).

In this way, ebvmiR-BART2-5p was detected in more children with IM and chronic high viral loads than in children who resolved the EBV infection. The same trend was observed among the EBV miRNAs expressed in the plasma and viral load. Several ebv-miRs were detected, including ebv-miRBART7-3p, ebv-miR-15, ebv-miR-9-3p, ebv-miR-11-3p, ebv-miR-1-3p, and ebv-miR-3-3p only in children with IM and with chronically high viral loads. Lytic ebvmiRs-BHRF1-2-3p and ebv-miR-1-1 (indicators of active viral replication) were only detected in children with MI [115]. Therefore, EBV-specific miRNA expression could represent a marker for monitoring the phase of infection in pediatric and EBV-infected kidney recipients.

Another vital point in viral infections in kidney transplantation is the Bk polyomavirus infection, which is usually a common asymptomatic infection in healthy people. However, in transplant patients with the continued use of immunosuppressants, which turns them into immunocompromised individuals, it can lead to nephropathy associated with polyomavirus and produce serious complications depending on its evolution [116].

It is known that the BK polyomavirus encodes two mature miRNAs, bkv-miR-B1-3p and bkv miR-B1-5p, which appear to regulate the life cycle of the virus itself [116] and appear to be involved in modulating and controlling viral replication, allowing the virus to evade the patient’s immune response.

Thus, it has been reported that patients with polyomavirus-associated nephropathy have or present a higher expression level than the healthy population [116]. In another study, bkv-miR-B1-5p and bkv-miR-BJ1-3p were prolific in urine samples associated with the level of urinary viral load but not in plasma samples in renal recipients with early-stage or late-stage infections [117]. Other studies in cultures show very limited conclusions in the clinical setting that must be resolved in the future [118].

Another important virus in kidney transplantation, which also produces quite common infections in the post-kidney transplant period in immunocompromised recipients due to immunosuppressants, is human cytomegalovirus (CMV) infection. This also encodes miRNA, which can influence the physiology and pathology of the viral infection itself. The viral miRNAs miR-UL112-5p, miR-US5-2-3p, miR-UL36, miR-US25-2-3p, and miR-UL22a encoded by CMV were detected in the saliva of kidney recipients, the miRNA miRUS5-2-3p being the one that appeared most frequently. These data seem to suggest an eventual association with CMV reactivation, and the measurement of the miRNA of the virus can serve to monitor the infection [119]. However, routine laboratory methods such as commercial Quantiferon CMV assays can help control CMV infection.

Table 5 shows a summary of the main findings on microRNA molecules in viral and bacterial infection in transplantation.

## 7. Future Directions and microRNAs in Therapeutic Approaches in Transplantation

Finally, validating miRNAs differentially expressed (DE) in rejection, complications, or transplant outcomes using additional mRNA microarray data from the Gene Expression Omnibus, as reported in earlier publications [8,120,121], may also be of actual interest.

Future research will be required to fully comprehend miRNAs’ role in graft rejection and how they might interact with other expression proteins [122]. However, it is necessary to mention that, in miRNA studies, a lack of proper standardization in data normalization methods still exists, nor does a gold standard and reliable endogenous control exist.

The miRNA target databases primarily contain theoretical interactions that have not been verified by experiments, which makes it exceedingly challenging to make an accurate interpretation. Validating these intriguing connections using the various sample types in each instance is also required. Despite these significant limitations, statistically sound assessments and analyses with positive implications for diagnosis have been published.

Considering this, emphasizing the significance of miRNA dysregulation is a frequent finding in these fundamental and clinical processes, particularly in tumoral processes that lead to cancer. It is crucial to talk about how other RNA transcripts, such as circRNAs and lncRNAs, can act as sponges for miRNAs in controlling many processes and diseases. In patients with cancer, inflammatory illnesses, or organ transplants, the regulation of the miRNA-mediated biological process can impact growth, invasion, modulation, immune activation or suppression, tolerance or rejection, and immunotherapy resistance. Given that miRNAs are regarded as “druggable targets,” further developments on their possible application ought to be debated, examined, confirmed, and ultimately put into practice for the benefit of our patients.

Regarding the eventual therapeutic role of these molecules, we have to consider that their small size makes them ideal for use as a healing agent. In this way, any mRNA or miRNA molecule can be modified to inhibit another specific miRNA or hinder or alter the miRNA–mRNA interaction. In this sense, this binding can lead to the inactivation of specific miRNAs, causing pathological processes, or if a specific miRNA has been poorly expressed or inhibited, its re-introduction will restore the modulation of an affected target gene [123,124].

In this way, artificial miRNAs (miRNA mimetic molecules) have been created to try to increase the expression of a specific beneficial miRNA or introduce short hairpin duplexes, similar to the pre-miRNA, into a target cell or an appropriate tissue. Apart from local injection into tissues, systemic administration can allow its development [123,124,125].

Thus, antisense oligonucleotides (ASOs) that seek to block miRNAs, specifically anti-miRNAs, can be developed [126]. If a miRNA is overexpressed, we can slow it down. In this sense, oligonucleotides have been artificially designed (“antagomirs”) as silencing elements of endogenous miRNAs in experimental work in mice [123,124,125,126].

miRNA sponges have also been artificially designed to inhibit several miRNAs with several binding sites and help sequester a family of miRNAs. Similarly, miRNA masks and erasers have also been designed to mask the miRNA binding site on its target (mRNA) or to use only two copies of the antisense sequence. Other gene-specific miRNA mimicking and miRNA masking antisense procedures and protocols have also been designed as eventual therapeutic targets [127,128].

How to ensure that all these inhibition, modulation, and regulation procedures for the activity of miRNAs specifically reach the specific organ or tissue is still a matter of further research and development.

To this end, extracellular vesicles are potentially being analyzed to administer, conduct, and deposit miRNAs or their inhibitors in the specific anatomical site we want to regulate [127]. They have been potentially involved therapeutically in kidney transplantation [128], communication, and interference between the allograft tissues and the immune system, promoting allorecognition, ischemia-reperfusion injury, or autoimmunity.

These vesicles, depending on the cell of origin that produces them, participate in improving complement activation or secreting complement inhibitors and preventing cell lysis, affect pro-coagulation and pro-thrombosis, promote endothelial survival and angiogenesis, and can induce rejection and/or autoimmunity with pro-coagulant and pro-inflammatory effects [128]. However, they can also promote immune tolerance [100]. These different studies show that transplanted patients with rejection show differential miRNA expression compared to tolerant transplants without rejection, indicating that these biomarkers could be potentially used to diagnose rejection in peripheral blood, urine samples, cells, or organ transplant recipients. Future assays, evaluations, and studies should be performed to corroborate these interesting results so that these can be applied in clinical practice to avoid invasive graft biopsies.

Table 6 shows a summary of the main findings on future directions and microRNAs in therapeutic approaches in transplantation.

In summary, this systematic review offers a comprehensive view of the role of microRNA (miRNA) molecules in various biological processes, focusing especially on their participation in transplantation and their potential as circulating biomarkers. In addition, the genesis and function of miRNAs are detailed, explaining their transcription, processing, and regulation mechanisms. Mature miRNAs can circulate extracellularly, especially in vesicles such as exosomes, with potential implications as biomarkers for pathological conditions. The stability of circulating miRNAs and their resistance to freeze–thaw cycles are highlighted, making them promising candidates as clinical biomarkers. The importance of standardizing collection, processing, transportation, and storage processes is emphasized for accurate analysis.

In the context of transplantation, kidney transplantation is specifically addressed, highlighting the role of miRNAs in predicting graft status, especially in acute rejection or delayed graft function. Several miRNAs, such as miR-150, miR-181, miR-191, miR-233, miR-346, miR-423-5p, and miR-574-3p, are implicated in different aspects of graft rejection, with possible connections to immunoregulatory processes and inflammatory pathways. The need for further research and validation in larger patient populations is recognized to establish the clinical utility of miRNAs in transplantation.

The role of miRNAs in B lymphocyte function and antibody production is delved into. This review concludes that miRNAs such as miR-150, miR-155, and miR-142-3p may play crucial roles in effector activities and regulatory networks that may control cell growth, isotype switch deficiency, and plasma cell development. However, the existing challenges in standardizing data normalization methods and the lack of reliable endogenous control in miRNA studies are recognized.

In conclusion, this systematic review highlights the potential of miRNAs as significant regulators in biological processes, valuable biomarkers in health and disease, and crucial players in the context of transplants. Although the field holds promise, the need for further research, standardization, and validation is highlighted to fully exploit the diagnostic and therapeutic implications of miRNAs in clinical practice.

## Figures and Tables

**Table 1:** Main findings on MicroRNA Molecules and Biological Functions.

Characteristics of miRNAs: miRNAs are non-coding endogenous RNA molecules with regulatory and modulatory roles. They prevent target mRNAs from being translated and have a length of 20–24 nucleotides. Studies estimate that miRNAs could regulate approximately 60% of the human transcriptome. Biological Processes Regulated by miRNAs: miRNAs play roles in critical biological processes such as cell cycle, differentiation, proliferation, and cell death. Dysregulation of specific miRNAs is associated with diseases like oncological, inflammatory, autoimmune, metabolic, and neurodegenerative conditions. Functional Mechanism of miRNAs: miRNAs bind to the complementary mRNA’s 3′-UTR region, inhibiting protein translation or promoting mRNA degradation. One miRNA can target hundreds of mRNAs, and vice versa, forming a complex network regulating genetic expression. Tissue-Specific Expression and Evolutionary Significance: miRNA expression patterns are tissue-specific and substantially conserved across species, emphasizing their significance for evolution. Over half of human mRNA transcripts are regulated by the 2600 mature miRNAs found in the human genome. Role of miRNAs in Diseases: Dysregulation of particular miRNAs is implicated in diseases, including oncological, inflammatory, autoimmune, metabolic, and neurodegenerative conditions. miRNAs act as intracellular regulators of gene expression and can have paracrine and endocrine effects. Interorgan Circulating Communication: miRNAs have been identified as interorgan circulating communication mediators. Hemodialysis does not eliminate circulating miRNAs, particularly in the context of kidney allograft transplant. Genesis and Function of miRNAs: miRNA transcription involves processes carried out by RNA polymerase II or III to primary miRNA (pri-mRNA). Cleavage by the Drosha-DGCR8 microprocessor complex leads to the formation of precursor pre-miRNA, which is further processed in the cytoplasm by RNase Dicer. Mature miRNA, loaded with the Argonaut (Ago2) protein into the RNA-induced silencing complex (RISC), guides the repression of target mRNA by various mechanisms. Extracellular Circulation of miRNAs: Mature miRNAs can travel from the cell cytoplasm into extracellular circulation or detect target mRNAs and cause mRNA silencing. Extracellular miRNAs, especially in physiological fluids and exosomes, are more stable than cellular miRNAs. Exosomal miRNAs, transported in vesicles, have implications in the pathophysiology and progression of cancer and may be clinically relevant. Principio del formulario

**Table 2:** Main findings on MicroRNAs as Circulating Biomarkers in Health and Disease.

Presence of miRNAs in Biofluids: miRNAs are found in biofluids, either packaged inside extracellular vesicles (exosomes and microvesicles) or bound to lipoproteins and ribonucleoproteins. Active cellular transport routes and passive leakage from damaged cells influence the composition of circulating miRNAs. Characteristics of Circulating miRNAs: Circulating miRNAs are stable in peripheral blood due to protection from endogenous RNases. They can withstand repeated freeze-thaw cycles, making them suitable for long-term sample storage. Tissue-Specific Expression Levels: Circulating miRNAs exhibit tissue-specific expression levels, adding to their significance as biomarkers. Quantification Methods: Quantitative measurement of miRNAs can be done using sensitive RT-qPCR assays. High-throughput methods like microarrays and RNA sequencing enable screening for specific miRNAs or analyzing larger miRNA panels. Applications in Clinical Settings: miRNAs serve as sensitive biomarkers for various metabolic and age-related disorders. Licensed diagnostic miRNA panels are available for cardiovascular illness, Alzheimer’s disease, thyroid, pancreatic, and breast cancer. Clinical Challenges and Considerations: Preanalytical errors, including factors like patient features (age, sex, body size), circadian rhythm, diet, and lifestyle, may impact miRNA expression. Standardization of collection, processing, transport, and storage processes is crucial to minimize variability. Time spent on the bench before processing can lead to hemolysis or blood cell leakage, altering miRNA expression. Analytical variability and the heterogeneity of commercially available tests reduce reproducibility and result in ambiguous outcomes. Standardization in Clinical Laboratories: The correct and mandatory standardization of miRNA testing in clinical laboratories is essential. Confounding elements should be thoroughly established and revised for accurate miRNA analysis in clinical settings.

**Table 3:** Main findings on MicroRNA Molecules and Their Role in Transplantation.

Focus of Kidney Allograft Transplantation Studies: Most studies on kidney allograft transplantation have concentrated on mRNA detection and expression in peripheral blood leukocytes, kidney biopsies, or urine samples. Role of miRNA Expression in Solid Organ Transplantation: Recent articles highlight the significance of miRNA expression in solid organ transplantation, suggesting a role in allograft acceptance or rejection. Promise of miRNA Expression Profiles in Predicting Kidney Graft Status: miRNA expression profiles show promise in predicting kidney graft status, including acute rejection or delayed graft function. Transcriptomics analysis aids in discovering expression profiles for patient stratification and differentiation of rejection types. Exploration of Urinary Exosomal miRNAs as Biomarkers: Research explores urinary exosomal miRNAs as potential biomarkers for acute rejection. Identification of candidate biomarkers involves NanoString-based profiling, meta-analysis, and literature review. Clinical Studies on Circulating miRNAs in Transplantation: Clinical studies evaluate circulating miRNAs (miR-10a, miR-92a, miR-155) as potential monitoring tools in heart allograft recipients. Investigation of miRNA expression levels in kidney transplant recipients with acute rejection episodes reveals specific miRNA alterations. Role of miR-150 in Immune Regulatory Processes: miR-150 plays a crucial role in immune regulatory processes, including B and T lymphocyte proliferation, activation, and apoptosis. Potential connection between miR-150-5p and transcription factor MBD6 in acute rejection episodes. Involvement of miR-181 and miR-191 in Rejection: miR-181 family and miR-191 show overexpression in patients with acute kidney rejection. miR-191 overexpression associated with bronchiolitis obliterans in lung transplantation and increased mortality risk in hematopoietic transplantation. Association of miR-223, miR-346, miR-423-5p, and miR-574-3p with Transplant Outcomes: miR-223 overexpression associated with acute rejection or tolerance biomarker in renal graft biopsies. Increased miR-346 linked to kidney rejection and correlated with severity of ischemia-reperfusion damage in heart transplantation. miR-423-5p linked with graft damage in kidney transplant recipients. Elevated miR-574-3p associated with delayed graft function in kidney recipients. Challenges and Need for Further Research: The role of miRNAs in organ transplantation requires continuous and exhaustive research, validation in multiple cohorts, and further studies for establishment in clinical routines. Principio del formulario

**Table 4:** Main findings on MicroRNA Molecules in B Cell, Humoral Rejection, and DSA Production.

Transcriptomics Studies in Tolerant Recipients: Increased expression of essential B lymphocyte genes like CD79B and CD20 observed in tolerant recipients. Decreased expression of CD79B, CD20, and TCL1A associated with patients experiencing acute rejection episodes. Elevated expression of cytokines (CXCL9 and CXCL10) by B lymphocytes in rejection processes after interaction with T lymphocytes. miRNAs in B Lymphocyte Subpopulations: Examination of miRNA expression in various B lymphocyte subpopulations, including germline naive B lymphocytes. miRNAs play a crucial role in effector activities and regulatory networks governing cell growth. Role of Specific miRNAs in B Cell Function and Antibody Production: miR-150-deficient animals exhibit increased antibody secretion in response to antigenic stimulation. Abnormalities in miR-155 lead to isotype change deficiency and poor plasma cell development. Overexpression of miR-142-3p in B cells from tolerant kidney transplant recipients, suggesting involvement in the TGF-signaling pathway. miRNA Expression in B Lymphocytes and Regulatory Pathways: miRNA expression in B lymphocytes, including naive and germinal center B lymphocytes, crucial in regulatory pathways of cell growth and effector functions. Additional Studies on Kidney Transplantation: miRNA expression changes associated with clinical complications like antibody-mediated rejection (AMR) in kidney transplantation. Up-regulation of let-7c, miR-28, miR-30d, miR-99b, miR-125a, miR-195, miR-374b, miR-484, miR-501, and miR-520c. Down-regulation of miR-29b and miR-885 associated with AMR in clinical complications.

**Table 5:** Main findings on MicroRNA Molecules in Viral and Bacterial Infection in Transplantation.

Bacterial Infections and miRNA Regulation: Gastrointestinal tract infections, mainly caused by uropathogenic Escherichia coli, are common in kidney transplant recipients. Immunosuppressant cyclosporine activates cells for adhesion and translocation of the pathogen. miRNA let-7i inhibits lipopolysaccharide-induced cell activation and down-regulates TLR4 expression. Anti-let-7i during renal treatment can protect patients from bacterial infections associated with cyclosporine. Viral Infections in Kidney Transplants: Epstein–Barr Virus (EBV) Infection: Pediatric kidney transplant patients infected with EBV show distinct viral miRNA profiles. EBV-specific miRNA expression, such as ebvmiR-BART2-5p, correlates with infectious mononucleosis and chronic high viral loads. EBV miRNAs, including ebv-miRBART7-3p, ebv-miR-15, and ebv-miR-9-3p, among others, serve as markers for infection monitoring. BK Polyomavirus Infection: Common asymptomatic infection in healthy individuals becomes a serious concern in immunocompromised transplant patients. BK polyomavirus encodes two mature miRNAs, bkv-miR-B1-3p and bkv miR-B1-5p, involved in regulating the virus life cycle. Elevated expression of bkv-miR-B1-5p associated with polyomavirus-associated nephropathy in kidney transplant patients. Limited conclusive findings in clinical settings that need resolution in the future. Human Cytomegalovirus (CMV) Infection: CMV, common in post-kidney transplant infections, encodes miRNAs influencing viral infection. Viral miRNAs, including miR-UL112-5p, miR-US5-2-3p, and miR-UL36, among others, detected in saliva of kidney recipients. miR-US5-2-3p is frequently detected, suggesting an association with CMV reactivation. Routine laboratory methods like commercial Quantiferon CMV assays aid in controlling CMV infection. Principio del formulario

**Table 6:** Main findings on Future Directions and microRNAs in Therapeutic Approaches in Transplantation.

Validation of Differentially Expressed (DE) miRNAs: Utilizing additional mRNA microarray data from the Gene Expression Omnibus (GEO) for validation. Existing publications highlight the importance of this approach in understanding rejection, complications, and transplant outcomes. Future Research Directions: Need for comprehensive research to fully comprehend miRNAs’ role in graft rejection. Exploration of interactions between miRNAs and other protein expressions. Challenges include lack of standardization in data normalization methods and absence of a reliable endogenous control. Significance of miRNA dysregulation in fundamental and clinical processes, especially in cancer. Role of Other RNA Transcripts: CircRNAs and lncRNAs act as sponges for miRNAs, influencing various processes and diseases. miRNA-mediated biological processes impact growth, invasion, modulation, immune activation, suppression, tolerance, rejection, and immunotherapy resistance. Therapeutic Potential of miRNAs: Consideration of their small size as an ideal feature for therapeutic use. Modification of mRNA or miRNA molecules to inhibit specific miRNAs or alter miRNA–mRNA interactions. Artificial miRNAs (miRNA mimetics) created to increase the expression of beneficial miRNAs. Antisense oligonucleotides (ASOs) developed as anti-miRNAs to block overexpressed miRNAs. Inhibition, Modulation, and Regulation Procedures: Development of antisense oligonucleotides (AMOs) and “antagomirs” for silencing endogenous miRNAs. Design of miRNA sponges, masks, erasers, and other gene-specific mimicking/masking antisense procedures for therapeutic targets. Ensuring specificity of these procedures to reach the intended organ or tissue requires further research and development. Extracellular Vesicles in Therapeutic Approaches: Analysis of extracellular vesicles for administering, conducting, and depositing miRNAs or their inhibitors. Potential therapeutic involvement in kidney transplantation and communication between allograft tissues and the immune system. Extracellular vesicles’ role in various physiological processes, immune tolerance, and potential diagnostic applications. Diagnostic Potential and Future Applications: Transplanted patients with rejection exhibit differential miRNA expression compared to tolerant transplants without rejection. Biomarkers like miRNAs could potentially diagnose rejection in peripheral blood, urine samples, cells, or organ transplant recipients. Future assays and studies needed for clinical application and to avoid invasive graft biopsies. Principio del formulario
